# Neuroimaging correlates of brain injury in Wilson’s disease: a multimodal, whole-brain MRI study

**DOI:** 10.1093/brain/awab274

**Published:** 2021-07-21

**Authors:** Samuel Shribman, Martina Bocchetta, Carole H Sudre, Julio Acosta-Cabronero, Maggie Burrows, Paul Cook, David L Thomas, Godfrey T Gillett, Emmanuel A Tsochatzis, Oliver Bandmann, Jonathan D Rohrer, Thomas T Warner

**Affiliations:** Reta Lila Weston Institute, UCL Queen Square Institute of Neurology, London WC1N 1PJ, UK; Dementia Research Centre, UCL Queen Square Institute of Neurology, London WC1N 3AR, UK; MRC Unit for Lifelong Health and Ageing, University College London, London WC1E 7HB, UK; Centre for Medical Image Computing, University College London, London WC1V 6LJ, UK; Biomedical Engineering and Imaging Sciences, King’s College London, London WC2R 2LS, UK; Tenoke Ltd, Cambridge CB2 0AH, UK; Reta Lila Weston Institute, UCL Queen Square Institute of Neurology, London WC1N 1PJ, UK; Department of Clinical Biochemistry, Southampton General Hospital, Southampton SO16 6YD, UK; Dementia Research Centre, UCL Queen Square Institute of Neurology, London WC1N 3AR, UK; Neuroradiological Academic Unit, UCL Queen Square Institute of Neurology, London WC1N 3BG, UK; Wellcome Centre for Human Neuroimaging, UCL Queen Square Institute of Neurology, London WC1N 3AR, UK; Department of Clinical Chemistry, Northern General Hospital, Sheffield S5 7AU, UK; UCL Institute of Liver and Digestive Health and Royal Free Hospital, London NW3 2PF, UK; Sheffield Institute of Translational Neuroscience, Sheffield S10 2HQ, UK; Dementia Research Centre, UCL Queen Square Institute of Neurology, London WC1N 3AR, UK; Reta Lila Weston Institute, UCL Queen Square Institute of Neurology, London WC1N 1PJ, UK

**Keywords:** Wilson’s disease, MRI, biomarker, atrophy, diffusion

## Abstract

Wilson’s disease is an autosomal-recessive disorder of copper metabolism with neurological and hepatic presentations. Chelation therapy is used to ‘de-copper’ patients but neurological outcomes remain unpredictable. A range of neuroimaging abnormalities have been described and may provide insights into disease mechanisms, in addition to prognostic and monitoring biomarkers. Previous quantitative MRI analyses have focused on specific sequences or regions of interest, often stratifying chronically treated patients according to persisting symptoms as opposed to initial presentation.

In this cross-sectional study, we performed a combination of unbiased, whole-brain analyses on T_1_-weighted, fluid-attenuated inversion recovery, diffusion-weighted and susceptibility-weighted imaging data from 40 prospectively recruited patients with Wilson’s disease (age range 16–68). We compared patients with neurological (*n* = 23) and hepatic (*n* = 17) presentations to determine the neuroradiological sequelae of the initial brain injury. We also subcategorized patients according to recent neurological status, classifying those with neurological presentations or deterioration in the preceding 6 months as having ‘active’ disease. This allowed us to compare patients with active (*n* = 5) and stable (*n* = 35) disease and identify imaging correlates for persistent neurological deficits and copper indices in chronically treated, stable patients.

Using a combination of voxel-based morphometry and region-of-interest volumetric analyses, we demonstrate that grey matter volumes are lower in the basal ganglia, thalamus, brainstem, cerebellum, anterior insula and orbitofrontal cortex when comparing patients with neurological and hepatic presentations. In chronically treated, stable patients, the severity of neurological deficits correlated with grey matter volumes in similar, predominantly subcortical regions. In contrast, the severity of neurological deficits did not correlate with the volume of white matter hyperintensities, calculated using an automated lesion segmentation algorithm. Using tract-based spatial statistics, increasing neurological severity in chronically treated patients was associated with decreasing axial diffusivity in white matter tracts whereas increasing serum non-caeruloplasmin-bound (‘free’) copper and active disease were associated with distinct patterns of increasing mean, axial and radial diffusivity. Whole-brain quantitative susceptibility mapping identified increased iron deposition in the putamen, cingulate and medial frontal cortices of patients with neurological presentations relative to those with hepatic presentations and neurological severity was associated with iron deposition in widespread cortical regions in chronically treated patients.

Our data indicate that composite measures of subcortical atrophy provide useful prognostic biomarkers, whereas abnormal mean, axial and radial diffusivity are promising monitoring biomarkers. Finally, deposition of brain iron in response to copper accumulation may directly contribute to neurodegeneration in Wilson’s disease.

## Introduction

Wilson’s disease is an autosomal-recessive disorder of copper metabolism caused by mutations in *ATP7B*.^1^ Impaired biliary excretion and reduced incorporation into apocaeruloplasmin leads copper to accumulate in the liver and other organs, including the brain.^2^ The clinical phenotype is variable and patients typically present with liver disease, a mixed movement disorder, psychiatric manifestations or any combination of these in childhood, adolescence or early adulthood.^3^ The international consensus classification therefore refers to ‘neurological’ presentations where a movement disorder or psychiatric features are present at onset and ‘hepatic’ presentations where these are absent.^4^

Chelating agents, such as penicillamine and trientine, facilitate the urinary excretion of copper and are used to ‘de-copper’ patients. Progressive deterioration and death were inevitable prior to their discovery.^5^ Outcomes for patients with neurological presentations, however, remain unpredictable and the majority have ongoing neurological symptoms or disability.^5^^,^^6^ Dosing regimens vary and monitoring is based on copper indices, such as the serum non-caeruloplasmin bound (‘free’) copper (NCC) and 24-h urinary copper output,^7–9^ which do not correlate with neurological severity at presentation.^10^

The neuropathological basis for Wilson’s disease appears to be primarily driven by the effects of excess copper in the brain.^11^^,^^12^ Histopathological changes including demyelination, reactive astrogliosis, central pontine myelinolysis, cavitation and abnormal iron deposition have been described, and both grey matter and white matter are affected.^13–17^ In parallel, a range of neuroimaging abnormalities including atrophy, white matter hyperintensities (WMHs), increased susceptibility and diffusion-related abnormalities are common.^18–21^ These may provide insights in to disease mechanisms, in addition to providing prognostic and monitoring biomarkers for neurological involvement.

Neuroimaging abnormalities in Wilson’s disease have largely been assessed using semi-quantitative measures in retrospective cohort studies. Recent quantitative MRI analyses, most of which have focused on specific sequences or regions of interest,^22–27^ have been informative, but unbiased whole-brain approaches across multiple modalities are needed to understand the clinical relevance of the complex neuroradiological findings seen in Wilson’s disease and determine their association with copper indices. An appreciation of the typical disease course is also required. Unlike most neurodegenerative disorders, Wilson’s disease mimics a protracted monophasic illness (if chelation therapy is initiated), which can therefore be modelled as a brain injury. A minority of chronically treated patients deteriorate due to non-adherence with chelation therapy leading to reaccumulation of copper and subsequent brain injury.^28^^,^^29^ Consideration as to whether there was a significant brain injury at onset and whether the insult is ongoing are important when interpreting neuroimaging abnormalities and cohorts need to be stratified accordingly.

We therefore aimed to determine the clinical relevance of neuroimaging abnormalities in Wilson’s disease by applying a combination of quantitative, whole-brain analyses on T_1_-weighted, fluid-attenuated inversion recovery (FLAIR), susceptibility-weighted and diffusion-weighted imaging (SWI and DWI) sequences from 40 prospectively recruited patients. We compared patients with neurological and hepatic presentations to determine the neuroradiological consequences of the initial brain injury. We then subcategorized patients according to their recent neurological status, classifying patients with recent neurological presentations or neurological deterioration associated with non-adherence as having ‘active’ neurological disease. This allowed us to identify imaging correlates for persistent neurological deficits and copper indices in chronically treated, stable patients and, secondarily, to compare patients with active and stable neurological disease to explore markers of evolving brain injury using our novel approach.

## Materials and methods

### Study population

Forty patients attended research visits for clinical assessments, venepuncture and neuroimaging between January and December 2019. Consecutive patients attending neurology, hepatology and metabolic outpatient clinics at the National Hospital for Neurology and Neurosurgery and Royal Free Hospital and members of the Wilson’s Disease Support Group UK research register were invited to participate. We included patients aged 16 years or over with a diagnosis of Wilson’s disease based on the Leipzig diagnostic criteria.^4^ Exclusion criteria were any unrelated medical or psychiatric illness that would interfere with completing assessments and pregnancy. All participants provided written informed consent and the study was approved by a regional ethics committee in October 2018 (18/NE/0279).

### Clinical and biochemical assessments

We interviewed participants and subsequently reviewed records, where necessary, to determine their presentation at the time of diagnosis according to the international consensus on the phenotypic classification of Wilson’s disease.^4^ Those who had initially presented with neurological or psychiatric symptoms were classified as having neurological presentations (*n* = 23). Those without these symptoms were classified as having hepatic presentations (*n* = 17), including asymptomatic individuals identified through family screening with previously abnormal liver function tests or hepatic imaging (*n* = 6). Participants were subcategorized according to their recent neurological status. Those with a neurological presentation (*n* = 2), or a documented deterioration in neurological function related to non-adherence (*n* = 3), in the preceding 6 months were classified as having active, as opposed to stable (*n* = 35), neurological disease.

The Unified Wilson’s Disease Rating Scale (UWDRS), which has been validated in several patient populations,^30^^,^^31^ was performed and the neurological examination (UWDRS-N) score was recorded. Subscores for specific neurological phenotypes were calculated based on individual items as described in Supplementary Table 1. Demographic and clinical characteristics, including evidence of cirrhosis (determined by previous imaging and histopathology results), disease duration (based on symptom onset) and treatments were documented. Serum caeruloplasmin and copper concentrations were measured using the immuno-turbidimetric test (Beckman Coulter) and inductively coupled plasma mass spectroscopy (NexION 300, PerkinElmer), respectively. Serum NCC concentrations were calculated.^7^

### Imaging acquisition, processing and analysis

MRI data were acquired on a Siemens Prisma 3 T system with a 64-channel head/neck coil (Siemens Healthcare). T_1_-weighted (structural), FLAIR, DWI and SWI data were collected using the pulse sequence parameters summarized in Supplementary Table 2. Data were visually inspected immediately after each acquisition to allow individual sequences to be repeated if movement artefacts were identified.

Group differences between patients with neurological and hepatic presentations, associations with UWDRS-N scores in stable patients, associations with NCC concentrations in stable patients and group differences between patients with active and stable disease were tested for each MRI sequence using the approaches described below. Age and sex were used as covariates in all imaging analyses and UWDRS-N was included as a covariate when comparing patients with active and stable neurological disease.

### T_1_-weighted (structural) imaging

Voxel-based morphometry (VBM) was performed using Statistical Parametric Mapping (SPM12, version 7771, http://www.fil.ion.ucl.ac.uk/spm).^32^ T_1_-weighted (structural) images were segmented into grey matter, white matter and CSF using standard procedures and spatially normalized using the fast-diffeomorphic image registration algorithm.^33^ Grey and white matter segments were transformed into MNI152 space (Montreal Neurological Institute, McGill University, Canada), modulated and smoothed using a Gaussian kernel with 8 mm full-width at half-maximum to create preprocessed grey matter tissue maps. All segmentations were visually checked for quality. The preprocessed tissue maps were fitted to factorial design analyses to identify group differences in grey matter volumes, and multiple regression analyses to identify associations with UWDRS-N and NCC. Total intracranial volume, calculated in SPM, was included as a nuisance covariate, in addition to age and sex.^34^ Threshold-free cluster enhancement was applied using the CAT12 toolbox with statistical thresholds set at *P* < 0.05 with family-wise-error (FWE) correction. A minimum cluster size of 20 voxels was set and statistical maps were overlaid onto the study-wise mean template.

We conducted a separate region of interest analysis to assess atrophy in specific subcortical structures. T_1_-weighted images were bias-corrected and parcellated using the geodesic information flow (GIF) pipeline,^35^ based on atlas propagation and label fusion. The brainstem was subsequently segmented using a customized version of a FreeSurfer module.^36^ The volume of eight subcortical region of interest including the caudate, putamen, pallidum, thalamus, amygdala, midbrain, pons and cerebellum, were extracted and expressed as a percentage of total intracranial volume, calculated in SPM. All segmentations were visually checked for quality. Linear regression was used to identify group differences in region of interest volumes or their associations with UWDRS-N or NCC. *P*-values for coefficients of interest both with and without false discovery rate (FDR) correction were calculated in R (version 3.6.0, http://www.R-project.org).

### FLAIR imaging

WMHs were segmented using Bayesian model selection, an automated lesion segmentation tool applied to rigidly co-registered T_1_-weighted (structural) and FLAIR sequences.^37^ A Gaussian mixture model with dynamically evolving number of components was fit to the data, modelling simultaneously healthy and non-expected observations. WMH-related measures were introduced to the model through subject-specific statistical atlases obtained using the GIF pipeline. After convergence, the model was used to select candidate lesion voxels whose aggregation in connected components was automatically classified as lesion or artefact. WMH segmentations were then visually inspected and flagged if there were significant segmentation errors. This quality control stage was used to make improvements to the automated WMH segmentation, thereby maximizing the number of usable segmentations.

The volume of WMHs within 40 anatomically defined regions was calculated for each participant.^38^ White matter was separated into four equidistant layers between the ventricular surface and the cortical grey matter/white matter interface. These were then divided into left and right frontal, temporal, parietal and occipital lobes using the GIF parcellation. The basal ganglia and infratentorial regions were considered separately. The volume of WMHs within each region was log_e_-transformed to reduce skewness. A linear regression model was used to identify group differences in the log_e_-transformed total volume of WMHs and the log_e_-transformed volume of WMHs within each region and their associations with UWDRS-N or NCC in stable patients. Total intracranial volume was included as a covariate of no interest, in addition to age and sex. *P*-values for coefficients of interest were calculated with FDR correction in R and these were summarized in bullseye plots to illustrate their anatomical distribution.^38^

### Diffusion-weighted imaging

The Functional MRI of the Brain Software Library (FSL, version 6.0.3, https://fsl.fmrib.ox.ac.uk/fsl) was used to pre-process DWI data prior to fitting the single tensor model, resulting in volumetric diffusion tensor imaging (DTI) data. DTI datasets were then analysed using tract-based spatial statistics (TBSS).^39^ Preprocessing included EDDY to correct for motion and eddy-currents with outlier replacement enabled. FUGUE was applied to correct for distortions using field maps. Tensors were fitted using DTIFIT and fractional anisotropy (FA), mean diffusivity (MD), axial diffusivity (AD) and radial diffusivity (RD) maps were generated, skeletonized and aligned using TBSS. Design matrices for identifying group differences or associations with UWDRS-N and NCC were generated using the general linear model. Finally, RANDOMISE was used to perform non-parametric permutation analyses based on each design matrix. Covariates were mean-centred and 10 000 permutations of the data were carried out. The threshold-free cluster enhancement (TFCE) algorithm was used to identify clusters of voxels with a FWE-corrected *P*-value < 0.05.^40^ Clusters of increased or decreased FA, MD, AD and RD were then overlaid onto a mask of the white matter skeleton (created using the mean skeletonized FA map) and the MNI152 template.

### Susceptibility-weighted imaging

Quantitative susceptibility maps (QSMs) were reconstructed from susceptibility-weighted images using a multi-scale dipole inversion-based pipeline for coil-combined, multi-gradient echo data in QSMbox (https://gitlab.com/acostaj/QSMbox).^41^ Preprocessing steps included unwrapping of complex 3D phase data using a discrete Laplacian method followed by background field removal using Laplacian boundary extraction and variable spherical mean filtering. All steps were applied using default settings. Whole-brain analyses were performed with the QSMexplorer pipeline (https://gitlab.com/acostaj/QSMexplorer).^42^ A study-wise space was created from T_1_-weighted sequences using advanced normalization tools. Bias-corrected magnitude images were then used to transform the quantitative susceptibility maps to the study-wise space. Absolute susceptibility maps smoothed with a 3-mm standard deviation (SD) 3D Gaussian kernel were used to identify group differences in susceptibility and the associations with UWDRS-N and NCC in stable patients. RANDOMISE was used to perform non-parametric permutation analyses based on each design matrix. Covariates were mean-centred and 10 000 permutations were performed. The grey and white matter segments generated in SPM12 were combined to mask the absolute maps. TFCE was enabled to identify clusters of voxels with a FWE-corrected *P*-value < 0.05. Clusters were then overlaid (for result visualization) onto the study-wise template.

### Statistical analysis

Group differences in demographic and clinical characteristics were tested in R. Normality was assessed using the Shapiro–Wilk test. Continuous variables were compared using unpaired *t*-tests and the Wilcoxon-signed rank test, frequencies were compared using the chi squared test and associations between continuous variables were assessed using Spearman's rank test. Graphs were created in Graphpad Prism 8 (version 8.4.1, https://www.graphpad.com/scientific-software/prism), unless otherwise stated.

### Data availability

Anonymized data are available on request to the corresponding author.

## Results

### Demographic and clinical characteristics

The cohort consisted of 23 patients with neurological presentations and 17 patients with hepatic presentations. Five patients with neurological presentations were subcategorized as having active neurological disease. This included two patients who had been diagnosed and started on chelation therapy in the preceding month and three chronically treated patients with a deterioration in neurological function documented at their last clinic appointment, all of whom volunteered that they had stopped taking chelation therapy.

The demographic and clinical characteristics are summarized in Table 1. There were no differences in age, sex, disease duration, evidence of cirrhosis, treatments or NCC concentrations between patients with neurological and hepatic presentation or between patients with active and stable disease. The mean age was 43 years (range 16–68) with a mean disease duration of 23 years. One participant had features of decompensated liver disease with ascites (without hepatic encephalopathy) at the time of their research visit. Three patients had previously undergone liver transplantation: two for acute liver failure and one for decompensated liver disease. UWDRS-N scores were higher in patients with neurological than hepatic presentations (22 versus 3, *P* < 0.001) and higher in patients with active than stable disease (48 versus 9, *P* = 0.001), as expected. The distribution of UWDRS-N scores and breakdown of individual participant scores by neurological phenotypes are depicted in Supplementary Fig. 1. There was no association between UWDRS-N scores or NCC concentration and disease duration.

**Table 1:** Demographics and clinical characteristics

	Hepatic	Neurological	Stable	Active
*n*	17	23	35	5
Mean age ± SD, years	42 ± 15	44 ± 14	44 ± 14	39 ± 17
Sex, female:male	8:9	12:11	19:16	1:4
Mean disease duration ± SD, years	20 ± 15	25 ± 16	24 ± 15	20 ± 20
Evidence of cirrhosis, yes:no	7:10	10:23	14:21	3:2
Treatment				
Penicillamine	9	17	23	3
Trientine	5	4	7	2
Zinc	1	0	1	0
Combination	0	1	1	0
Transplantation	2	1	3	0
Median NCC [IQR], μmol/l	1.7 [1.4–2.3]	1.7 [1.4–2.6]	1.7 [1.4–2.3]	2.0 [1.8–3.1]
Median UWDRS-N score [IQR]	3 [0–4]	22 [14–37]	9 [3–17]	48 [40–51]

One participant with a neurological presentation who was considered to have stable disease declined to undergo MRI during the research visit and a participant with a hepatic presentation had T_1_-weighted (structural) acquisitions only. Two further patients with neurological presentations did not complete the DWI acquisitions.

### Atrophy

**Figure 1:**
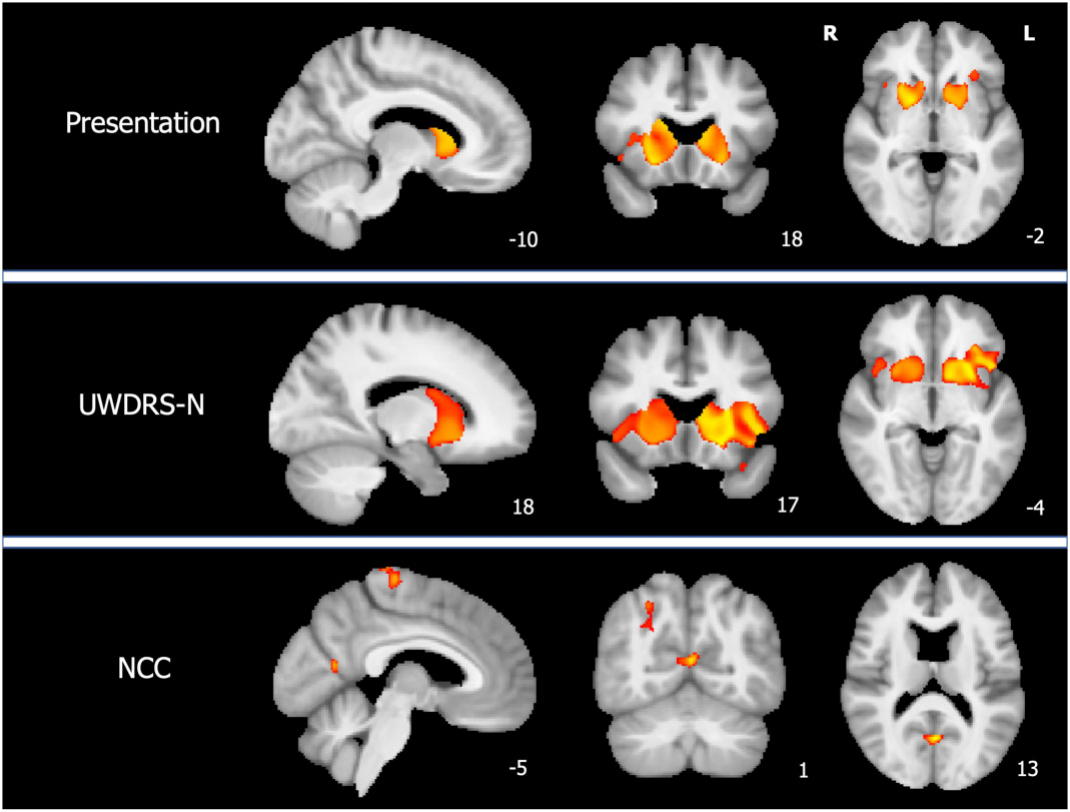
**VBM results.** Tissue maps showing clusters where grey matter volumes are lower in patients with neurological than hepatic presentations, decrease with increasing UWDRS-N scores and decrease with increasing NCC concentrations for FWE-corrected *P*-values < 0.05. Clusters are overlaid onto the study-wise mean template. For visualization purposes one slice in each of the sagittal (*x*), coronal (*y*) and axial (*z*) planes was selected and MNI coordinates are provided. L = left; R = right.

VBM results are shown in Fig. 1 and Supplementary Table 3. Region of interest analyses are summarized in Table 2. Patients with neurological presentations had lower grey matter volumes than patients with hepatic presentations in the bilateral caudate, putamen and nucleus accumbens, left orbitofrontal and central opercular cortices and right anterior insula cortex with VBM analysis. The region of interest analyses showed that caudate, putamen, pallidum, thalamus, midbrain, pons and cerebellum volumes were significantly lower in patients with neurological than hepatic presentations with FDR-corrected *P*-values < 0.05.

**Table 2:** Group differences and associations with region of interest volumes

	Hepatic	Neurological	*P*	UWDRS-N	*P*	NCC	*P*	Stable	Active	*P*
Caudate	0.45 ± 0.05	0.36 ± 0.05	<0.001***	−1.5 × 10^−5^	0.004**	6.6 × 10^−5^	0.38	0.41 ± 0.06	0.35 ± 0.08	0.62
Putamen	0.61 ± 0.07	0.49 ± 0.05	<0.001***	−2.2 × 10^−5^	0.002**	2.4 × 10^−5^	0.82	0.54 ± 0.09	0.46 ± 0.07	0.86
Pallidum	0.27 ± 0.03	0.21 ± 0.03	<0.001***	−0.9 × 10^−5^	0.003**	3.9 × 10^−5^	0.39	0.24 ± 0.04	0.20 ± 0.04	0.79
Thalamus	0.77 ± 0.07	0.67 ± 0.09	<0.001***	−1.4 × 10^−5^	0.05	8.5 × 10^−5^	0.41	0.71 ± 0.09	0.61 ± 0.15	0.63
Amygdala	0.24 ± 0.02	0.24 ± 0.02	0.90	−0.2 × 10^−5^	0.33	−2.2 × 10^−5^	0.35	0.23 ± 0.02	0.23 ± 0.03	0.08
Midbrain	0.42 ± 0.04	0.34 ± 0.07	<0.001***	−1.3 × 10^−5^	0.02*	−4.9 × 10^−5^	0.55	0.37 ± 0.06	0.29 ± 0.12	0.74
Pons	0.99 ± 0.10	0.80 ± 0.15	<0.001***	−2.9 × 10^−5^	0.03*	−5.0 × 10^−5^	0.79	0.87 ± 0.14	0.75 ± 0.28	0.67
Cerebellum	9.61 ± 0.75	8.83 ± 1.09	0.007**	−14.1 × 10^−5^	0.03*	−6.9 × 10^−5^	0.93	9.33 ± 0.79	9.16 ± 2.04	0.82

The mean % of total intracranial volume ± SD in each group and correlation coefficients for associations with UWDRS-N and NCC in stable patients are shown. Results where *P* < 0.05 after FDR correction are highlighted in bold.

* *P* < 0.05.

** *P* < 0.01.

*** *P* < 0.001.

UWDRS-N scores in stable patients were associated with a similar pattern of reduced grey matter volumes extending to orbitofrontal and anterior insula cortices bilaterally and were associated with reduced caudate, putamen, pallidum, midbrain, pons and cerebellum volumes for region of interest analyses. In contrast, NCC concentrations in stable patients were negatively correlated with grey matter volumes in scattered cortical areas including the left precentral gyrus, right lateral occipital cortex and bilateral precuneus. There were no associations between NCC concentrations and the volume of any region of interest in stable patients and no differences between patients with active and stable disease on VBM or region of interest analyses.

### White matter hyperintensities

The log_e_-transformed total volume of WMHs did not differ between patients with hepatic and neurological presentations (916 versus 1384 mm^3^, *P* = 0.12) and was higher in patients with active disease than patients with stable disease (6126 versus 953 mm^3^, *P* < 0.001). There were no associations between the total volume of WMHs and UWDRS-N scores (β = 0.0, *P* = 0.63) or NCC concentrations (β = −0.1, *P* = 0.68) in stable patients.

**Figure 2:**
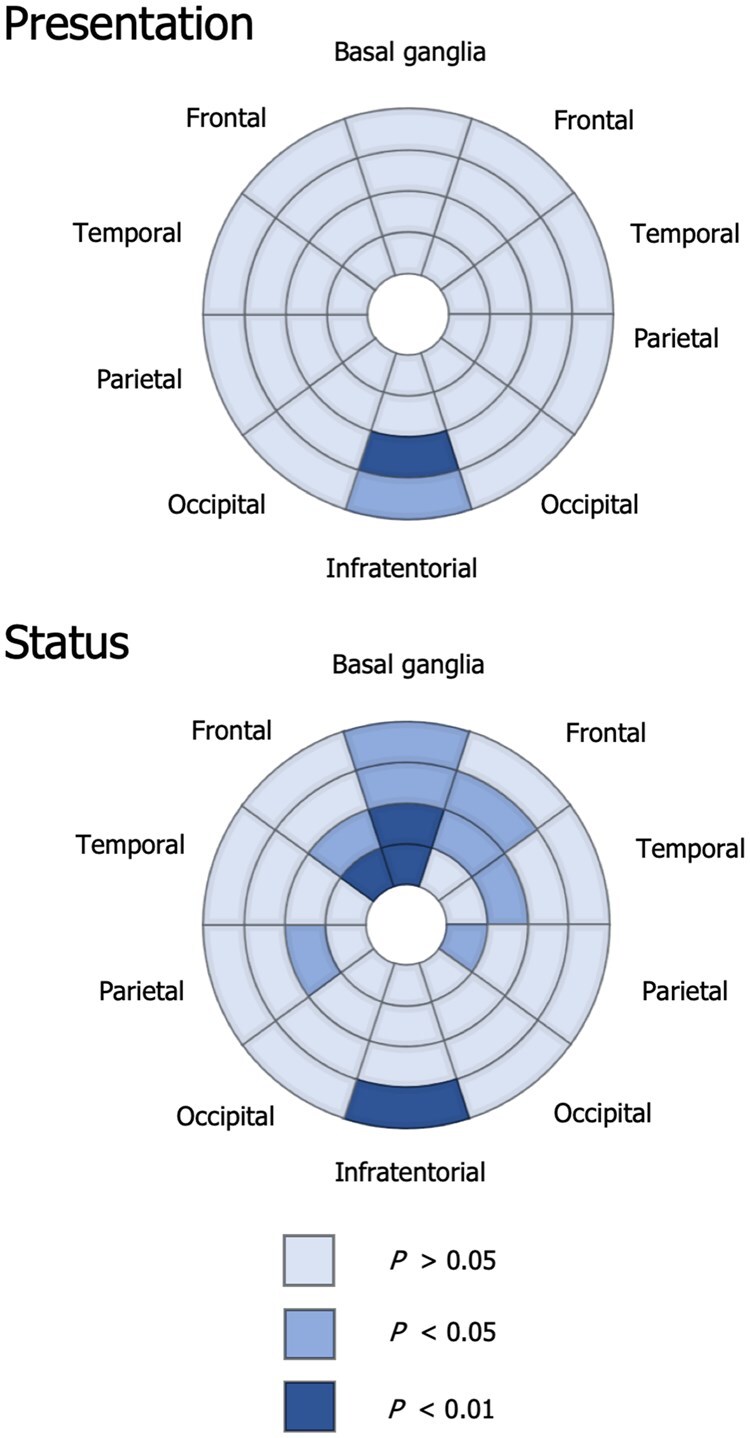
**Automated lesion segmentation results.** Bullseye plots showing FDR-corrected *P*-values for significant group differences in the volume of WMHs between patients with neurological and hepatic presentations and between patients with active and stable neurological disease. Each sector is subdivided into four layers corresponding to more central or peripheral locations.

Bullseye plots demonstrating group differences in the log_e_-transformed volume of WMHs within individual regions are shown in Fig. 2. Patients with neurological presentations had higher infratentorial lesion volumes than patients with hepatic presentations. This finding did not persist when excluding patients with active disease on *post hoc* testing. Patients with active disease had higher lesions volumes in the basal ganglia, periventricular frontal, temporal and parietal white matter and infratentorial regions. There were no associations between lesion volumes within individual regions and UWDRS-N scores or NCC concentrations in stable patients (data not shown).

### Diffusion tensor imaging

**Figure 3:**
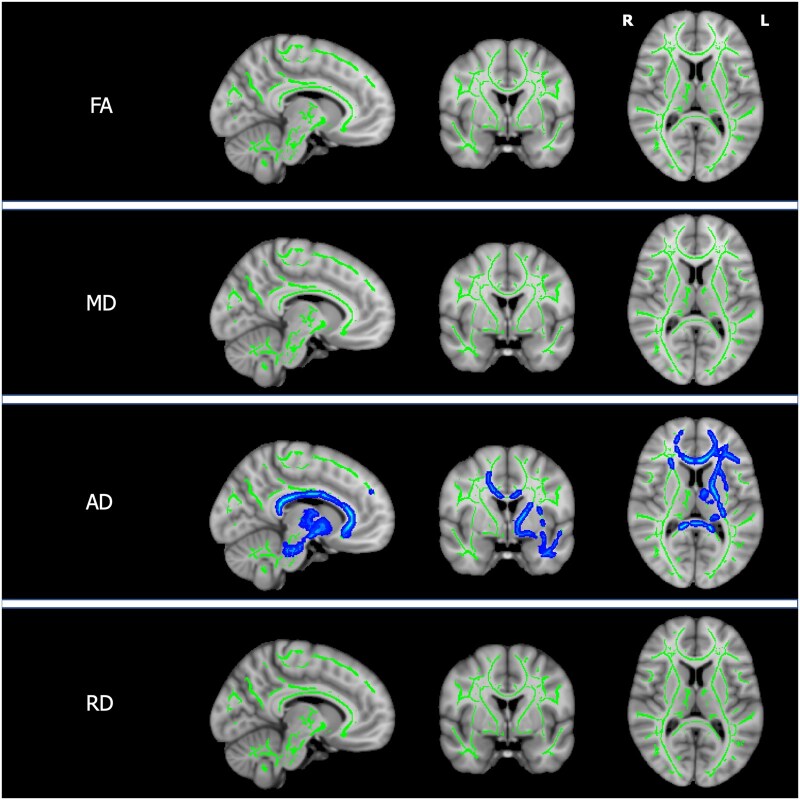
**TBSS for UWDRS-N scores.** Tissue maps showing significant correlations between diffusion indices (FA, MD, AD and RD) in white matter tracts and UWDRS-N scores in stable patients for FWE-corrected *P*-values < 0.05. Tracts with negative correlations (blue) are overlaid onto the white matter skeleton (green) and MNI152 template. For visualization purposes one slice in each of the sagittal, coronal and axial planes was selected with MNI coordinates −9, 0, 10. L = left; R = right.

**Figure 4:**
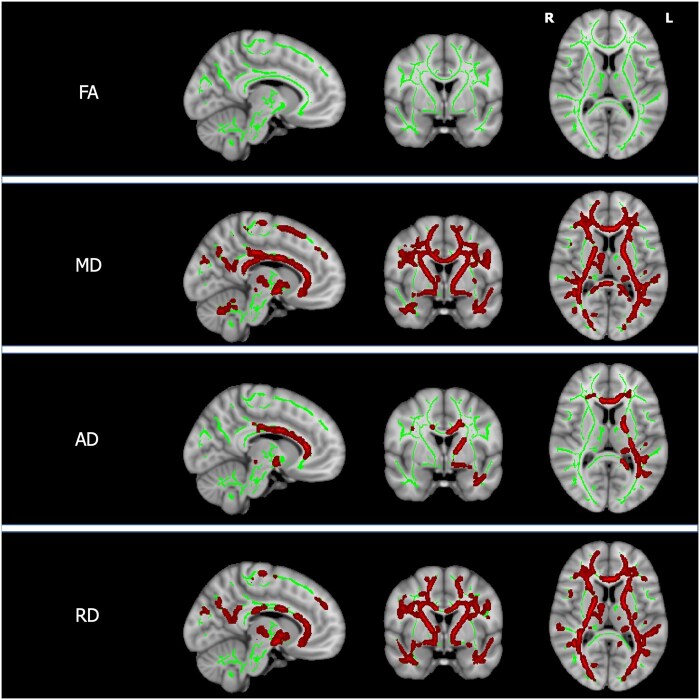
**TBSS for NCC concentration.** Tissue maps showing significant correlations between diffusion indices (FA, MD, AD and RD) in white matter tracts and NCC concentrations in stable patients for FWE-corrected *P*-values < 0.05. Tracts with positive correlations (red) are overlaid onto the white matter skeleton (green) and MNI152 template. For visualization purposes one slice in each of the sagittal, coronal and axial planes was selected with MNI coordinates −9, 0, 10. L = left; R = right.

**Figure 5:**
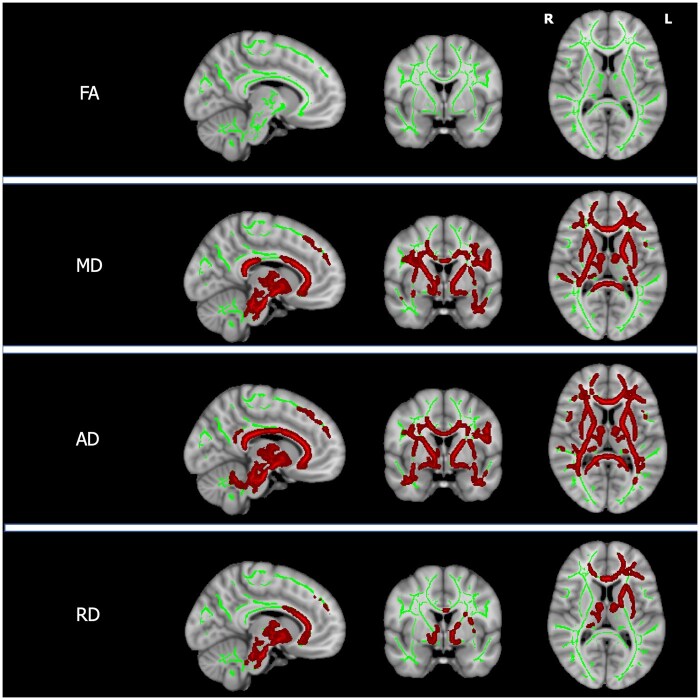
**TBSS for disease status.** Tissue maps showing white matter tracts where FA, MD, AD and RD significantly differ between patients with active and stable disease for FWE-corrected *P*-values < 0.05. Tracts where DTI indices are higher in patients with active disease (red) are overlaid onto the white matter skeleton (green) and MNI152 template. For visualization purposes one slice in each of the sagittal, coronal and axial planes was selected with MNI coordinates −9, 0, 10. L = left; R = right.

There were no differences in FA, MD, AD or RD when comparing the white matter tracts of patients with neurological and hepatic presentations. Increasing UWDRS-N scores in stable patients were associated with reduced AD in left internal and external capsules, anterior thalamic radiation, uncinate fasciculus and cerebral peduncle and, bilaterally, in the corpus callosum and forceps minor as shown in Fig. 3 and Supplementary Fig. 2. There were no associations between UWDRS-N scores and FA, MD or RD. Increasing NCC concentrations in stable patients were associated with increased MD and RD throughout the white matter and increased AD in the left anterior thalamic radiation, inferior longitudinal fasciculus and corpus callosum as shown in Fig. 4 and Supplementary Fig. 3. Patients with active disease had increased MD and AD throughout the white matter, with the exception of the inferior longitudinal fasciculus bilaterally, as shown in Fig. 5 and Supplementary Fig. 4. RD was increased in the left internal and external capsules, anterior thalamic radiation and forceps minor and the cerebral peduncle and corpus callosum bilaterally. There were no significant FA associations with NCC concentrations or group differences between patients with active and stable disease.

### Quantitative susceptibility mapping

**Figure 6:**
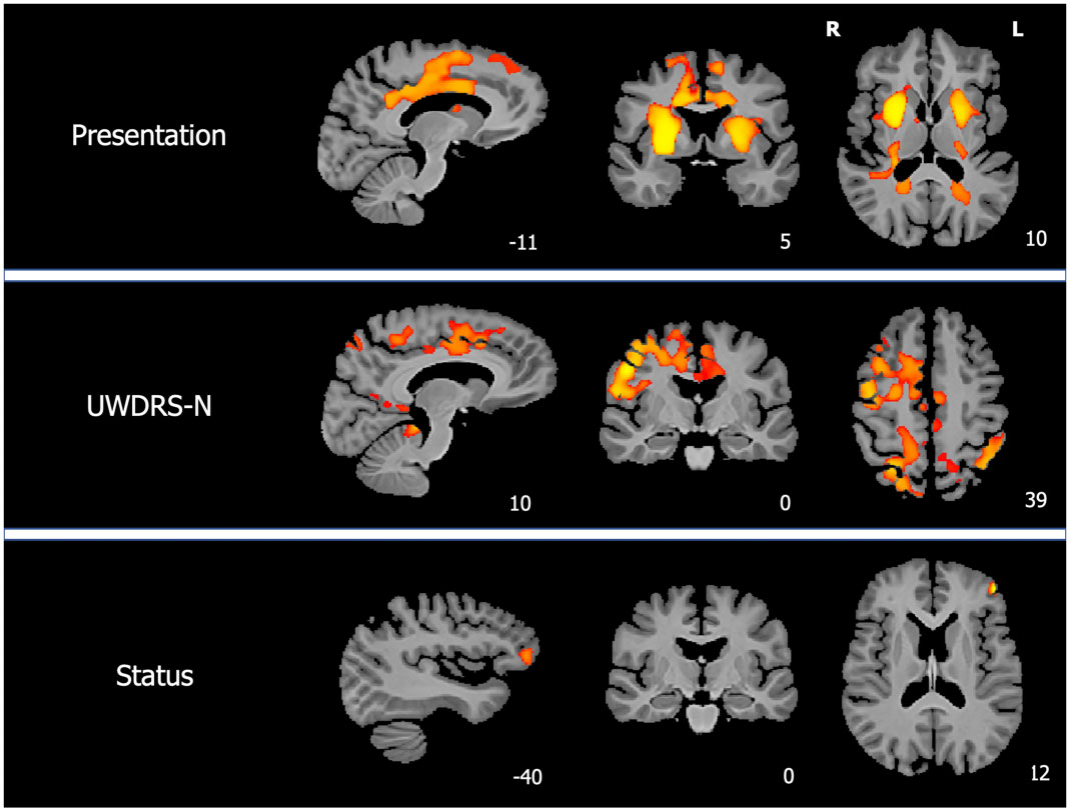
**Whole-brain QSM results.** Clusters where absolute susceptibility is significantly higher in patients with neurological than hepatic presentations, increases with increasing UWDRS-N scores and is higher in patients with active than stable disease are shown for FWE-corrected *P*-values < 0.05. Clusters are overlaid onto the study-wise template. For visualization purposes one slice in each of the sagittal (*x*), coronal (*y*) and axial (*z*) planes was selected and coordinates are provided. L = left; R = right.

Group differences in absolute susceptibility and associations with UWDRS-N scores in stable patients are shown in Fig. 6 and Supplementary Figs 5–7. Patients with neurological presentations had increased absolute susceptibility in the bilateral putamen, cingulate and medial frontal cortices compared to patients with hepatic presentations. Clusters extended into regions of white matter including the internal and external capsules, corpus callosum and, to a lesser extent, corona radiata. Increasing UWDRS-N scores in stable patients were associated with increased susceptibility in scattered cortical areas within the bilateral frontal, parietal and occipital lobes, cingulate cortices, right cerebellar hemisphere but not the basal ganglia. A few of these clusters extended into adjacent white matter tracts. There were no associations between NCC concentrations and absolute susceptibility and patients with active disease had increased susceptibility in one cluster within the left frontal pole compared with patients with stable disease. On *post hoc* testing, this cluster appeared to be driven by the chronically treated, non-adherent patients.

## Discussion

The combination of whole-brain quantitative neuroimaging methods applied in our cohort offers the most comprehensive analysis of the relationship between clinico-biochemical characteristics and neuroimaging abnormalities in Wilson’s disease to date. In classifying participants according to the presence of neurological symptoms at onset and recent neurological status, we have identified neuroimaging correlates of the initial brain injury by comparing patients by neurological and hepatic presentations and found associations with disease severity and copper indices in chronically treated, stable patients across four MRI sequences. The distinct patterns of diffusion and susceptibility abnormalities seen when comparing patients by presentation as opposed to those associated with UWDRS-N scores in chronically treated, stable patients justifies our approach to cohort stratification and emphasizes that neuroradiological abnormalities need to be taken in context of the initial brain injury. In combination with our observations of brain atrophy and WMHs, our data have implications for disease mechanisms and how specific MRI abnormalities could be used as prognostic or monitoring biomarkers in Wilson’s disease.

Several large retrospective studies have previously demonstrated that cortical, brainstem and cerebellar atrophy, assessed by visual inspection, is common in newly diagnosed patients with neurological presentations.^18^^,^^19^^,^^43^ In a preceding VBM study, Stezin *et al*.^23^ compared 10 adolescent patients with neurological presentations with age-matched controls and identified reduced grey matter volumes in the bilateral caudate, putamen and areas within the frontal and temporal cortices, in addition to the left thalamus and cerebellum. Segmentation-based studies have subsequently confirmed that basal ganglia and brainstem volumes are reduced in Wilson’s disease^24^^,^^44^^,^^45^; however, reports on the association with neurological severity were conflicting.^22^^,^^25^ Dusek *et al*.^46^ recently used deformation and surface-based morphometry to demonstrate Wilson’s disease causes widespread brain atrophy most pronounced in central structures, including large regions of white matter, and that increasing neurological severity is associated with volume loss in the putamen, pallidum and surrounding white matter.

Using a combination of VBM and region of interest analyses, we found that caudate, putamen, pallidum, thalamus, brainstem and cerebellum volumes were lower in patients with neurological than hepatic presentations. A similar pattern of atrophy was seen when testing the association with UWDRS-N scores in chronically treated, stable patients suggesting that atrophy in these regions not only occurs in relation to the initial brain injury but also represents a clinically relevant neuroimaging end-point in Wilson’s disease. We also identified reduced volumes in the orbitofrontal and anterior insula cortices using VBM, which has not previously been described in Wilson’s disease. These cortical areas have an important role in emotional processing and decision-making,^47^^,^^48^ which can be affected in neurological presentations.^49^^,^^50^

Interestingly, neurological involvement was not associated with more widespread cortical volume loss despite cortical atrophy being common in neurological presentations. We did, however, find that NCC concentrations in stable patients were inversely associated with grey matter volumes in scattered regions within the frontal and occipital cortices. This supports the observation made by Smolinski *et al*.^22^ that NCC concentrations correlate with a measure of total brain volume in newly diagnosed patients and suggests that, while increasing circulating copper concentrations are associated with cortical atrophy, neurological outcomes are more closely associated with subcortical atrophy. This is perhaps unsurprising given the established role of the basal ganglia, brainstem and cerebellum in the aetiology of other movement disorders, but raises questions about the clinical significance of cortical atrophy and how this relates to cognitive impairment and psychiatric features in Wilson’s disease.

T_2_-weighted hyperintensities in the basal ganglia, thalamus and/or brainstem are characteristic of Wilson’s disease.^18^^,^^43^^,^^51^^,^^52^ There appears to be an association between the overall lesion load measured using semi-quantitative scales and neurological severity at the time of diagnosis,^43^^,^^51^ and a reduction in lesion load is seen after initiation of chelation therapy in most patients,^53–55^ although lesions may persist or progress despite clinical improvement.^56–58^ We have performed the first quantitative analysis of the volume and distribution of WMHs in Wilson’s disease using an automated lesion segmentation tool. Our findings underscore that patients with active neurological disease not only have basal ganglia and infratentorial hyperintensities, but also have periventricular white matter lesions within the frontal, temporal and parietal lobes. However, the lack of association between the volume of WMHs and neurological severity in stable patients, and the lack of significant differences between patients with neurological and hepatic presentations, is unexpected. It suggests that while subcortical WMHs are seen in patients with active neurological disease, the extent to which they persist does not correlate with neurological outcomes.

There are several putative mechanisms underlying WMHs in Wilson’s disease including demyelination, oedema, gliosis, tissue necrosis and cavitation.^13–17^ Our data support the idea that some lesions, and therefore some of the pathological processes responsible for these lesions, are reversible whereas others are not. The distribution of lesions, extending to more diffuse areas of periventricular white matter, also emphasizes the importance of recognizing white matter pathology and potential consequences for network dysfunction in Wilson’s disease.

DTI abnormalities including decreases in FA and increases in MD have previously been demonstrated in the white matter of newly diagnosed patients using region of interest analyses.^20^^,^^27^^,^^59–61^ Associations between a neurological symptom score and increased MD in the anterior limb of the internal capsule and between a measure of disability and increased MD in frontal white matter have also been reported.^59^ In one of the few previous prospective neuroimaging studies on Wilson’s disease, Lawrence *et al*.^27^ used TBSS to analyse whole-brain changes in white matter diffusivity in 35 patients with neurological presentations, 17 of whom were drug-naive; FA increased and MD, AD and RD decreased over 24 months of chelation therapy; however, the relationship with clinical features was untested. Hu *et al*.^45^ recently confirmed that patients with Wilson’s disease have decreased FA and increased MD, RD and AD in various association and limbic tracts and identified correlations between AD and an event-based prospective memory task. The association between DTI indices and copper indices has, to our knowledge, only been investigated in a single study that measured FA and fibre volumes in white matter tracts and found no association with total serum copper, urinary copper output or CSF copper.^62^

We used TBSS to describe abnormalities in FA, MD, AD and RD in white matter in our cohort. While we found no differences between patients with neurological and hepatic presentations, there was a clear association between increasing UWDRS-N scores and decreasing AD in the subcortical white matter tracts of stable patients. In contrast, we found increasing NCC concentrations were associated with increased MD, AD and RD in subcortical white matter tracts of stable patients. In patients with active disease, although small in number, there was higher MD, AD and RD in subcortical white matter tracts than patients with stable disease. Increases in RD were more widespread when testing the association with NCC concentrations, whereas increases in AD were more widespread when testing the difference between patients with active and stable disease.

We suggest that the association between increasing neurological severity and decreasing AD seen in chronically treated patients reflects axonal loss, i.e. white matter atrophy. Smolinski *et al*.^22^ observed that total white matter volume was inversely associated with UWDRS-N in newly diagnosed patients and there is supporting evidence from a mouse model of Wallerian degeneration that axonal loss correlates with reductions in AD.^63^ We are wary about attributing pathological processes to other DTI abnormalities in the absence of disease-specific histopathological correlation. Increases in membrane permeability, fibre reorganization, destructions of intracellular compartments and glial alterations could all plausibly affect diffusion of water molecules in unanticipated directions.^64^ Irrespective of the mechanism, our observations on the association between NCC concentrations and diffusivity suggest that either the disease is not adequately controlled in some stable patients, or that copper can disrupt white matter diffusivity in stable patients without causing neurodegeneration.

In contrast to previous DTI studies,^25^^,^^27^^,^^59^ we did not identify any abnormalities in FA in the white matter tracts in our analyses. We suspect that this relates to the small number of newly diagnosed patients in our cohort, who may have more pronounced changes in white matter diffusivity. Our data do, however, highlight the importance of considering absolute measures of diffusivity, such as AD and RD, separately in Wilson’s disease. We have demonstrated distinct patterns of abnormalities in these parameters related to neurological severity, copper indices and disease activity that may be missed or underappreciated with an overreliance on FA and MD, which are essentially functions of AD and RD. Our data also support observations in Alzheimer’s disease that early neuropathological processes can be associated with changes in white matter diffusivity that are proportional along each semi-principal axis and therefore do not alter FA.^65^

Abnormal susceptibility has previously been demonstrated in the basal ganglia, thalamus, red nucleus and substantia nigra of patients with Wilson’s disease using several region of interest-based approaches.^21^^,^^46^^,^^66^^,^^67^ Susceptibility values in these areas are higher in patients with neurological than hepatic presentations,^25^^,^^26^^,^^67^^,^^68^ and correlate with UWDRS scores in some reports but not others.^46^^,^^69^^,^^70^ In a post-mortem 7 T MRI study, Dusek *et al*.^16^ demonstrated that increased susceptibility in the caudate, putamen and pallidum corresponds to increased iron but not copper content. They also suggested that cortical iron deposition might be increased in patients with neurological presentations. Cortical SWI abnormalities have, however, only been described in a case series of two patients with neurological presentations.^71^

Using whole-brain quantitative susceptibility mapping, we found that patients with neurological presentations have increased absolute susceptibility in the putamen, cingulate and medial frontal cortices relative to patients with hepatic presentations. Several of the clusters we identified extended into subcortical white matter tracts, including the internal and external capsules, and the corpus callosum. Interestingly, increasing UWDRS-N scores were associated with increased absolute susceptibility in widespread areas of cortical grey matter. We suspect that increased absolute susceptibility in cortical regions represents abnormal iron deposition, as opposed to other causes of paramagnetic or diamagnetic mineralization. Loss of myelin has been shown to cause increasing (less negative) susceptibility in white matter but leads to decreasing (less positive) susceptibility in grey matter.^72^ This would not, therefore, explain our findings in cortical regions. Analysis of R2* maps may help delineate whether clusters we identified in white matter when comparing patients with neurological and hepatic presentations represent iron or loss of myelin, but histopathological studies will ultimately be required.

These observations on cortical susceptibility might have important implications for our understanding of the pathophysiological basis for neurological involvement in Wilson’s disease. Taken in isolation, they are consistent with the idea that iron deposition is an epiphenomenon in neurodegeneration and occurs as a secondary consequence of copper accumulation and/or neuronal injury. However, we did not find an association between NCC concentrations and cortical susceptibility, or between increasing neurological severity and cortical atrophy in our cohort. The reason why only some patients with Wilson’s disease develop neurological involvement remains unclear despite concerted efforts to identify correlations with genotype and genetic modifiers in the *ATP7B* interactome.^73–75^ Our findings may suggest that a tendency to mishandle iron in response to copper accumulation (or cerebral caeruloplasmin deficiency) contributes more directly to neurodegeneration. It may mean the ability to maintain iron metabolism successfully confers a tolerance to the toxic effects of copper in the brain. This would explain the lack of significant differences between copper indices in patients with neurological and hepatic presentations,^10^ and how patients with hepatic presentations can have markedly increased cerebral copper content but not develop neurological symptoms.^12^^,^^16^

The mechanism through which iron deposition occurs in Wilson’s disease is unknown; however, ATP7B is required for the biosynthesis of caeruloplasmin, a copper-dependent ferroxidase that plays an important role in iron metabolism. Caeruloplasmin expression in Purkinje neurons is decreased in *Atp7b* knockout mice and acaeruloplasminaemia, caused by loss of function mutations in the *CP* gene, leads to a brain iron accumulation disorder.^76^ Our hypothesis on the relationship between iron deposition and neurodegeneration and the extent to which caeruloplasmin contributes to dysregulation of brain iron metabolism needs to be tested in animal and cell-based models of Wilson’s disease.

Overall, our findings have implications for how MRI can be used to develop prognostic and monitoring biomarkers for neurological involvement in Wilson’s disease. Guidelines suggest using clinical examination alone to monitor response to treatment.^7^^,^^8^ Several semi-quantitative scales have been devised to provide a pragmatic approach to measuring neuroradiological abnormalities in a clinical setting.^20^^,^^43^^,^^77^ Dusek *et al*.^78^ recently proposed a sophisticated system that includes an acute toxicity score, based on WMHs, and a chronic damage score, based on atrophy and SWI abnormalities. In a validation study, UWDRS-N scores correlated with the chronic damage score but not the acute toxicity score in newly diagnosed patients.^78^ The acute toxicity score improved whereas the chronic damage score deteriorated with treatment and neither baseline scores were able to predict outcomes UWDRS-N scores at 24 months. Of the four regions used to measure atrophy, our VBM and TBSS results suggest that central and brainstem atrophy, measured in the aforementioned study using third ventricle width and antero-posterior midbrain diameter, respectively, are more likely to predict neurological outcomes than cortical or cerebellar atrophy, measured by visual inspection. Further characterization between these measures and regional brain volumes would help validate our findings. We did not observe an association between UWDRS-N scores and SWI abnormalities in the basal ganglia and suggest that measuring atrophy alone in subcortical regions is more likely to provide a robust prognostic biomarker for predicting response to chelation therapy, or stratifying patients in clinical trials.

Our observations on WMHs, specifically the lack of association between these and UWDRS-N scores in chronically treated patients, highlight the limitations of using FLAIR or T_2_-weighted imaging to monitor treatment response. While it is reassuring to see lesions resolve, our results suggest that persistence of WMHs may not necessarily indicate treatment failure. Some of our most striking findings relate to DTI abnormalities in white matter, particularly the seemingly bidirectional relationship AD shows in active and stable disease and the association between diffusivity and NCC concentrations. We suggest that DTI may provide insights as to whether a given patient has reversible or irreversible neurological involvement. Further longitudinal studies are required.

The main limitations of this study are the small number of patients with active disease and lack of healthy controls. The former reflects the challenge in recruiting patients with rare diseases for prospective studies and any absence of differences between patients with active and stable disease should be interpreted with care. The active group also included a combination of newly diagnosed and chronically treated patients. *Post hoc* analyses suggest that the isolated SWI abnormality we identified in patients with active disease were primarily driven by chronically treated, non-adherent patients, and this group should ideally be considered separately. The lack of healthy controls limits the extent to which our findings inform us about neuroimaging abnormalities in Wilson’s disease more broadly. Patients classified as having hepatic presentations based on the absence of neurological or psychiatric symptoms may have subclinical neuroimaging abnormalities and so differences between patients with neurological and hepatic presentations are not equivalent to those between patients and healthy controls. We also recognize that a binary classification for neurological involvement based on findings at presentation is an oversimplification and there is likely to be a continuum of neurological involvement. Some patients with hepatic presentations develop neurological signs in the long-term leading to heterogeneity in the hepatic group.^79^ Several authors take an alternative approach, classifying patients according to the presence of persisting neurological features,^25^^,^^44^ and this may account for some disparate results in the literature. Paradoxical worsening, which is poorly understood but occurs in 11% of patients, may also need to be considered.^80^ Prospectively measuring UWDRS-N scores prior to and after initiating treatment would provide a more detailed characterization of the initial brain injury in Wilson’s disease but was not feasible in this study. Finally, NCC concentrations calculated using serum caeruloplasmin and serum copper may be less accurate than direct measurement using, for example, strong anion exchange chromatography coupled with tandem mass spectrometry.^81^

To conclude, we have provided evidence that a specific pattern of predominantly subcortical grey matter atrophy involving the basal ganglia, brainstem, cerebellum and anterior insula is associated with brain injury in Wilson’s disease by comparing patients with neurological and hepatic presentations and, unlike the volume and distribution of WMHs, significantly correlates with neurological outcomes. Using TBSS, we have shown that increasing neurological severity is also associated with subcortical white matter atrophy and that increasing NCC concentrations are associated with abnormal diffusivity throughout the white matter of chronically treated, stable patients. Our QSM data suggest that patients with neurological presentations have abnormal iron deposition in the putamen, cingulate and medial frontal cortices relative to patients with hepatic presentations, whereas increasing neurological severity is associated with increased susceptibility in widespread cortical areas. This suggests a role for brain iron metabolism in neurodegeneration in Wilson’s disease. Prospective studies of newly diagnosed patients that carefully characterize clinical progress in parallel with DTI indices as brain injuries evolve with chelation therapy will be required to understand better the role of white matter diffusivity as a biomarker for monitoring neurological involvement in Wilson’s disease.

## Supplementary Material

awab274_Supplementary_Data

## Abbreviations

AD: axial diffusivity
DTI: diffusion tensor imaging
FA: fractional anisotropy
MD: mean diffusivity
NCC: non-caeruloplasmin-bound copper
RD: radial diffusivity
SWI: susceptibility-weighted imaging
TBSS: tract-based spatial statistics
UWDRS-N: Unified Wilson’s Disease Rating Scale-Neurological examination
VBM: voxel-based morphometry
WMHs: white matter hyperintensities
