# Supplementary material for: Neuroimaging correlates of brain injury in Wilson’s disease: a multimodal, whole-brain MRI study
Source: Brain. 2021 Jul 21;145(1):263–75. doi: 10.1093/brain/awab274 (PMC8967100; doi:10.1093/brain/awab274)

Supplementary Table 1. Neurological phenotypes and corresponding UWDRS-N items

Phenotype	Item
Speech	Speech (10)
Dystonia	Oromandibular dystonia (11A)
	Cervical dystonia (22)
	Arm and hand dystonia (23)
	Posture – truncal dystonia (25A)
	Gait – leg dystonia (26A)
Parkinsonism	Facial expression – hypomimia (11B)
	Finger taps (15)
	Rapid alternating movements (16)
	Leg agility (20)
	Posture – parkinsonism (25C)
	Gait – parkinsonism (26C)
Tremor	Tremor at rest (12)
	Head tremor (13)
	Tremor in arms – postural (18A)
	Tremor in arms – wing-beating (18B)
	Postural tremor in legs (21)
Ataxia	Finger-to-nose test (19)
	Posture – ataxia of stance (25B)
	Gait – ataxia (26B)
Chorea	Chorea (27)
Rigidity	Rigidity (14)
Writing	Handwriting (17)

The UWDRS item numbers proposed by Leinweber et *al.*[30] are included in parentheses.

Supplementary Table 2. MRI acquisition parameters

	3D T1	3D FLAIR	DWI	3D SWI	B0 fieldmap
Pulse sequence	MPRAGE	IR-SPACE	Double refocused PGSE-EPI	3D gradient echo	2D gradient echo
Voxel resolution (mm^3)	1.1 x 1.1 x 1.1	1.0 x 1.0 x 1.0	2.5 x 2.5 x 2.5	1.0 x 1.0 x 1.0	3.0 x 3.0 x 3.0
Matrix size	256 x 256 x 208	256 x 256 x 192	96 x 96 x 59	256 x 192 x 176	64 x 64 x 55
Field of view (mm)	282 x 282 x 229	256 x 256 x 192	240 x 240 x 148	256 x 192 x 176	192 x 192 x 165
Orientation	Sagittal	Sagittal	Axial	Axial	Axial
Phase-encoding direction	A >> P	A >> P	A >> P	R >> L	R >> L
Echo time, TE (ms)	2.93	403	90	4.94/9.88/14.82/19.76/24.70	4.92/7.38
Recovery time, TR (ms)	2000	4800	7300	30	688
Flip angle (degrees)	8	Variable	-	15	60
Acquisition bandwidth (Hz/Px)	240	751	1578	280/260/260/260/260	260
Parallel imaging (GRAPPA acceleration factor)	2	3	2	3	None
Total scan time	5 min 6 sec	4 min 54 se	8 min 47 sec	4 min 9 sec	1 min 31 sec
Other sequence specific parameters	Inversion time, IR, 850 ms	Inversion time, TI, 1650 ms<br>SPACE turbo factor 243	Twice-refocused 2D multi-slice SE-EPI readout, b = 1000 s/mm^2 for diffusion encoding along 64 orientations. Five interspersed b = 0 s/mm^3 scans.	Partial Fourier 6/8<br>Monopolar readout<br>Flow compensation for first echo.	2D multi-slice

Supplementary Table 3. VBM statistics

**Analysis**	**Anatomical region**	**Cluster size [illegible]**	**TFCE**	$P\text{-}_{FWE}$	$P\text{-}_{uncorr}$	**x**	**y**	**z**
**Presentation**	Right subcortical	2952	213390	0.009	0.000	22	15	-10
	Left subcortical	2180	145797	0.013	0.000	-10	16	9
	Left orbitofrontal	128	53891	0.028	0.001	-32	32	-4
	Left operculum	21	44711	0.037	0.002	-56	-6	6
**UWDRS-N**	Left subcortical	6808	2443	0.005	0.001	-27	10	-8
	Right subcortical	3420	2040	0.011	0.001	21	20	-9
**NCC**	Bilateral precuneus	240	1500	0.039	0.001	0	-63	14
	Left pre-central	247	1477	0.041	0.001	-8	-26	70
	Right lateral occipital	118	1443	0.045	0.000	27	-60	48

Supplementary Figure 1. UWDRS-N breakdown of neurological phenotypes Each row represents an individual participant. Patients with hepatic presentations are included and six patients had a UWDRS-N score of zero. Phenotypes are denoted by colours. Speech, rigidity and writing subscores relate to multiple phenotypes and are therefore grouped separately.

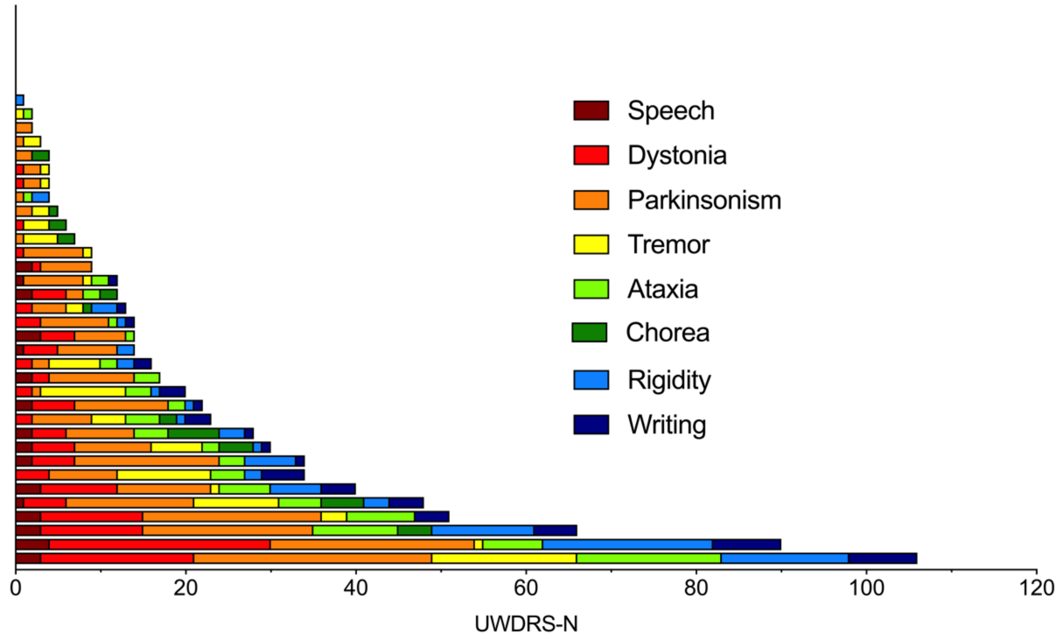

Supplementary Figure 2. TBSS for UWDRS-N scores Tissue map showing correlations between AD and UWDRS-N scores in stable patients for FWE-corrected P values < 0.05. Tracts with negative correlations (blue) are overlaid onto the white matter skeleton (green) and MNI152 template. Axial slices are shown.

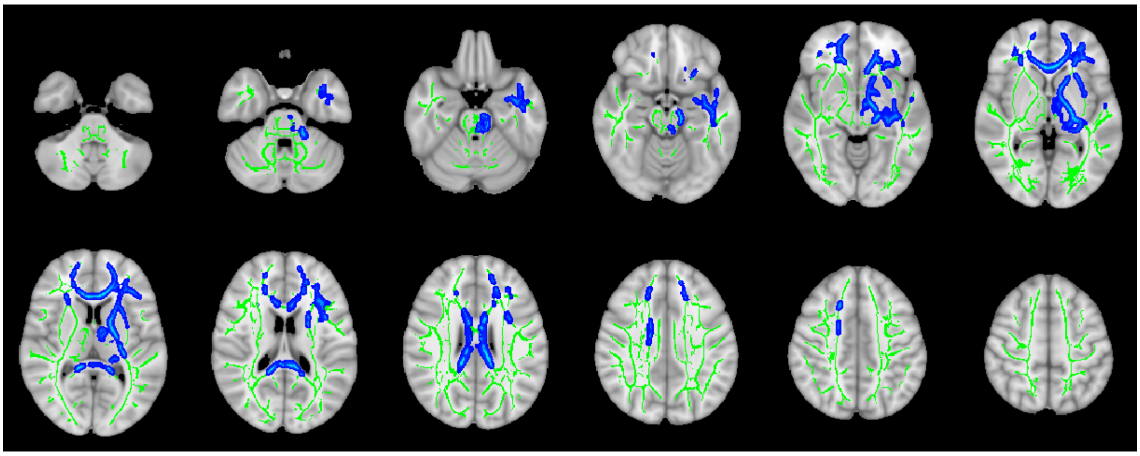

Supplementary Figure 3. TBSS for NCC concentrations Tissue map showing correlations between MD, AD and RD and NCC concentrations in stable patients for FWE-corrected P values < 0.05. Tracts with positive correlations (red) are overlaid onto the white matter skeleton (green) and MNI152 template. Axial slices are shown.

A) MD

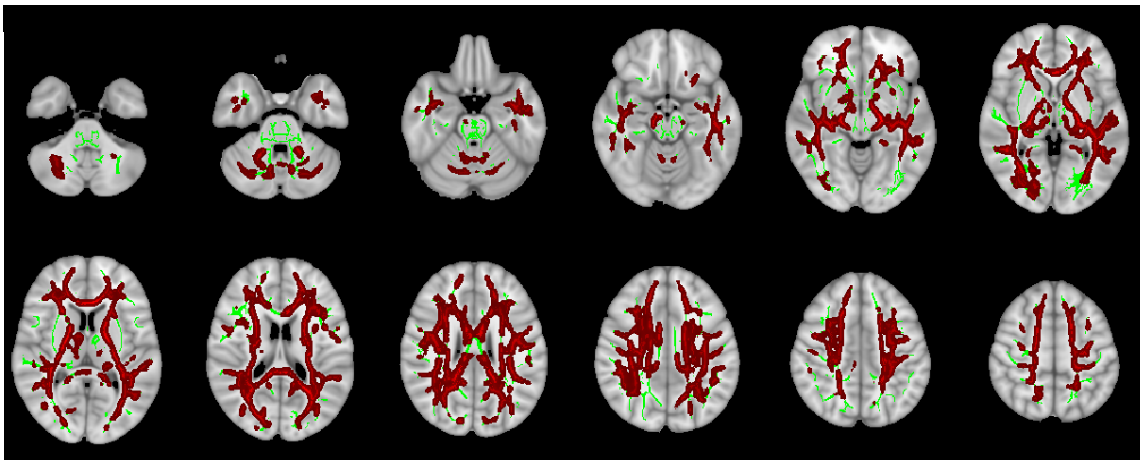

B) AD

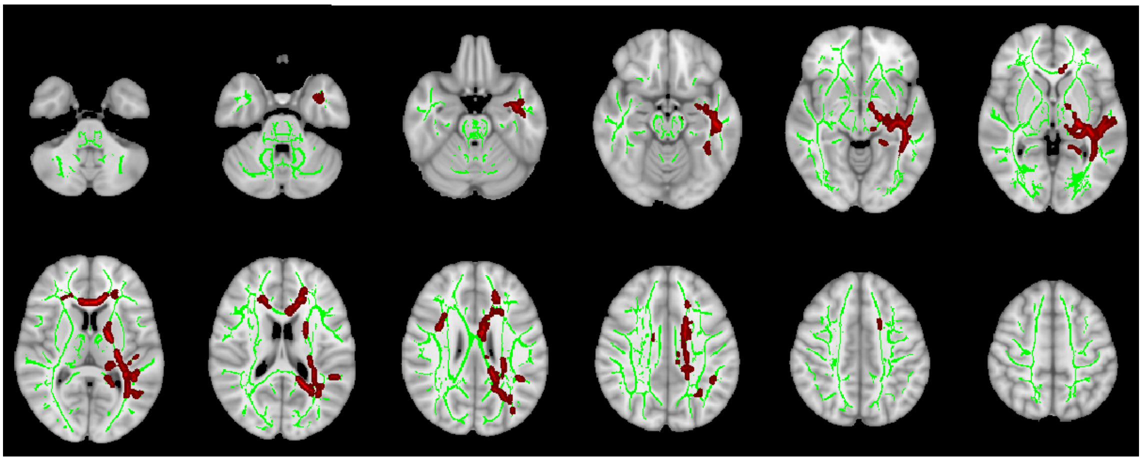

C) RD

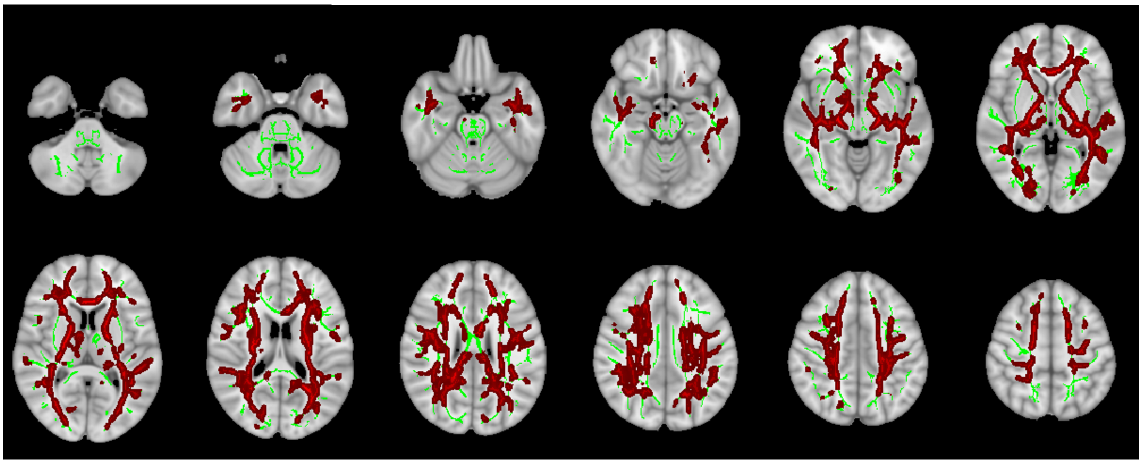

Supplementary Figure 4. TBSS for disease status Tissue maps showing differences in MD, AD and RD between patients with active and stable disease for FWE-corrected P values < 0.05. Tracts where indices are higher with active disease (red) are overlaid onto the white matter skeleton (green) and MNI152 template. Axial slices are shown.

A) MD

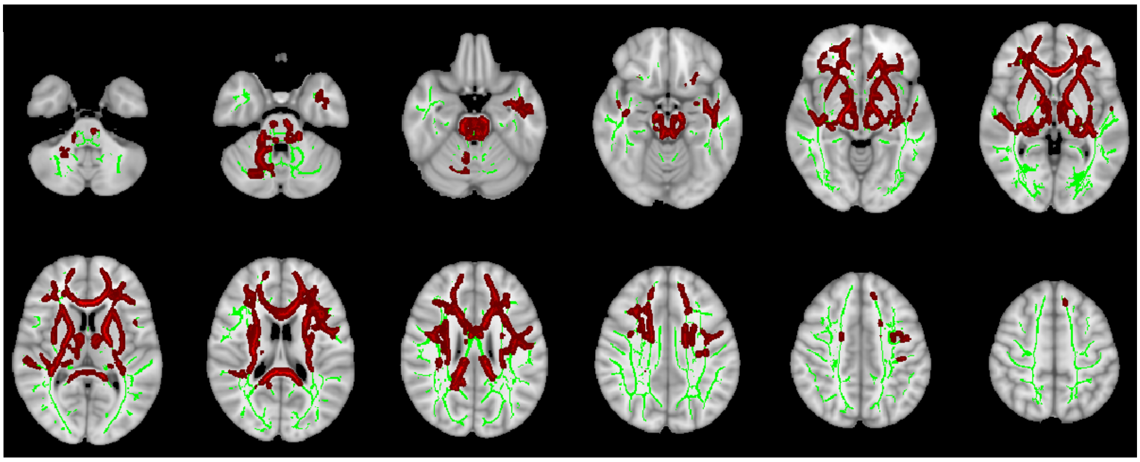

B) AD

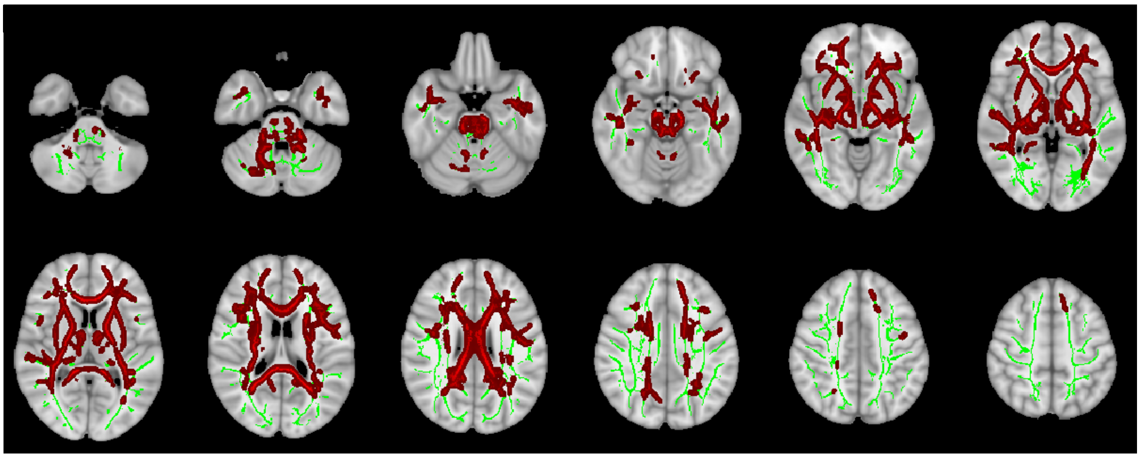

C) RD

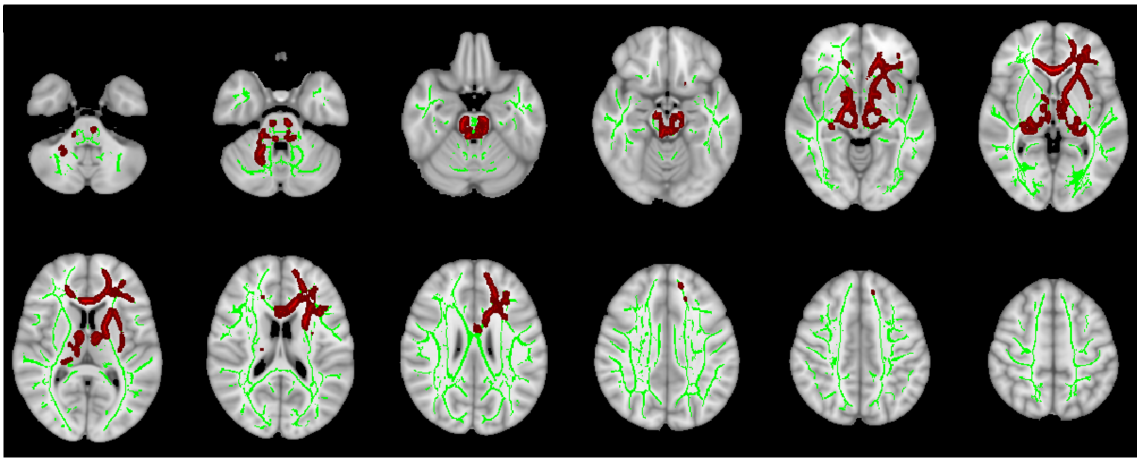

Supplementary Figure 5. Whole-brain QSM for presentations Clusters where absolute susceptibility is significantly higher in patients with neurological than hepatic presentations are shown for FWE-corrected P values < 0.05. Clusters are overlaid onto the study-wise template. Axial slices are shown.

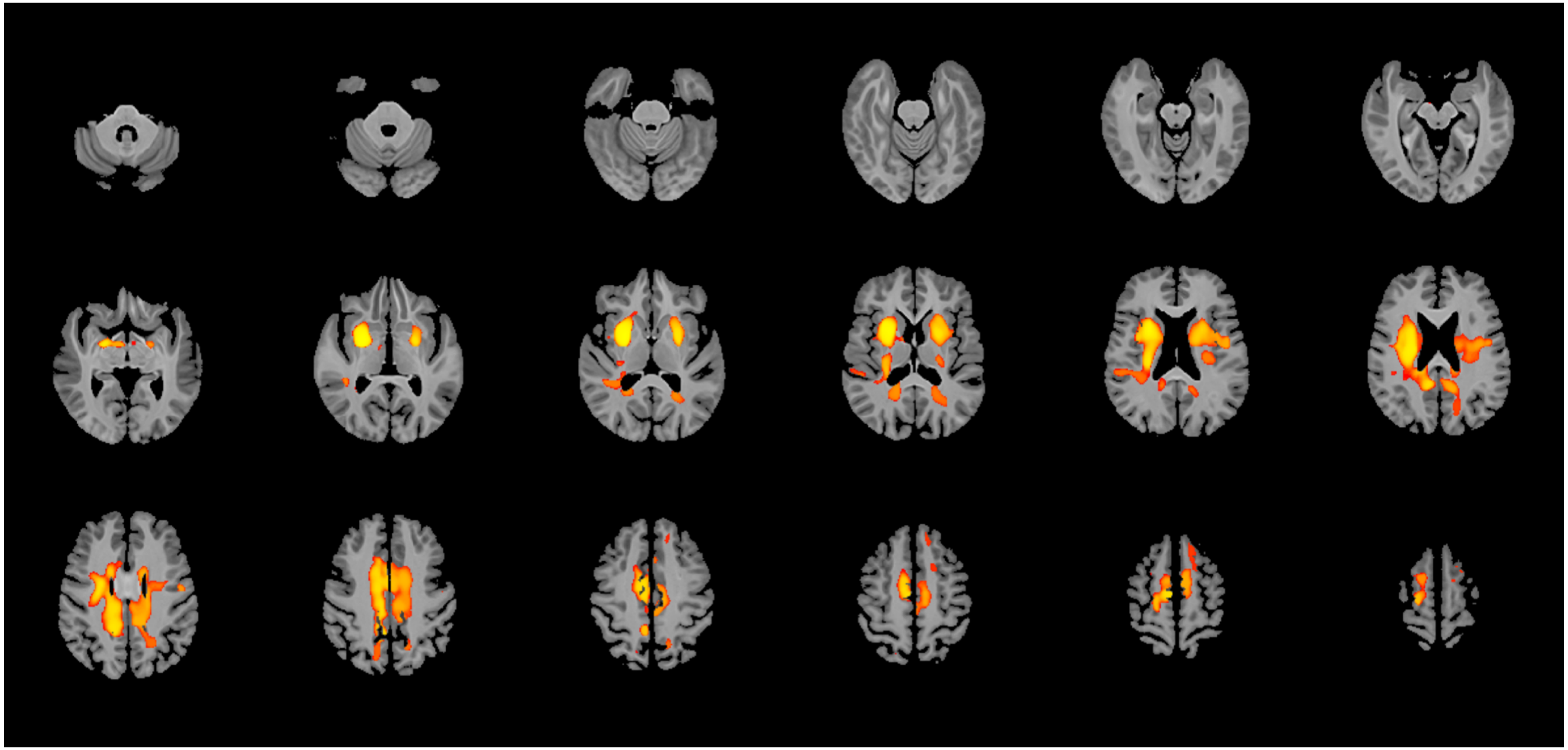

Supplementary Figure 6. Whole-brain QSM for UWDRS-N scores Clusters where absolute susceptibility increases with increasing UWDRS-N scores are shown for FWE-corrected P values < 0.05. Clusters are overlaid onto the study-wise template. Axial slices are shown.

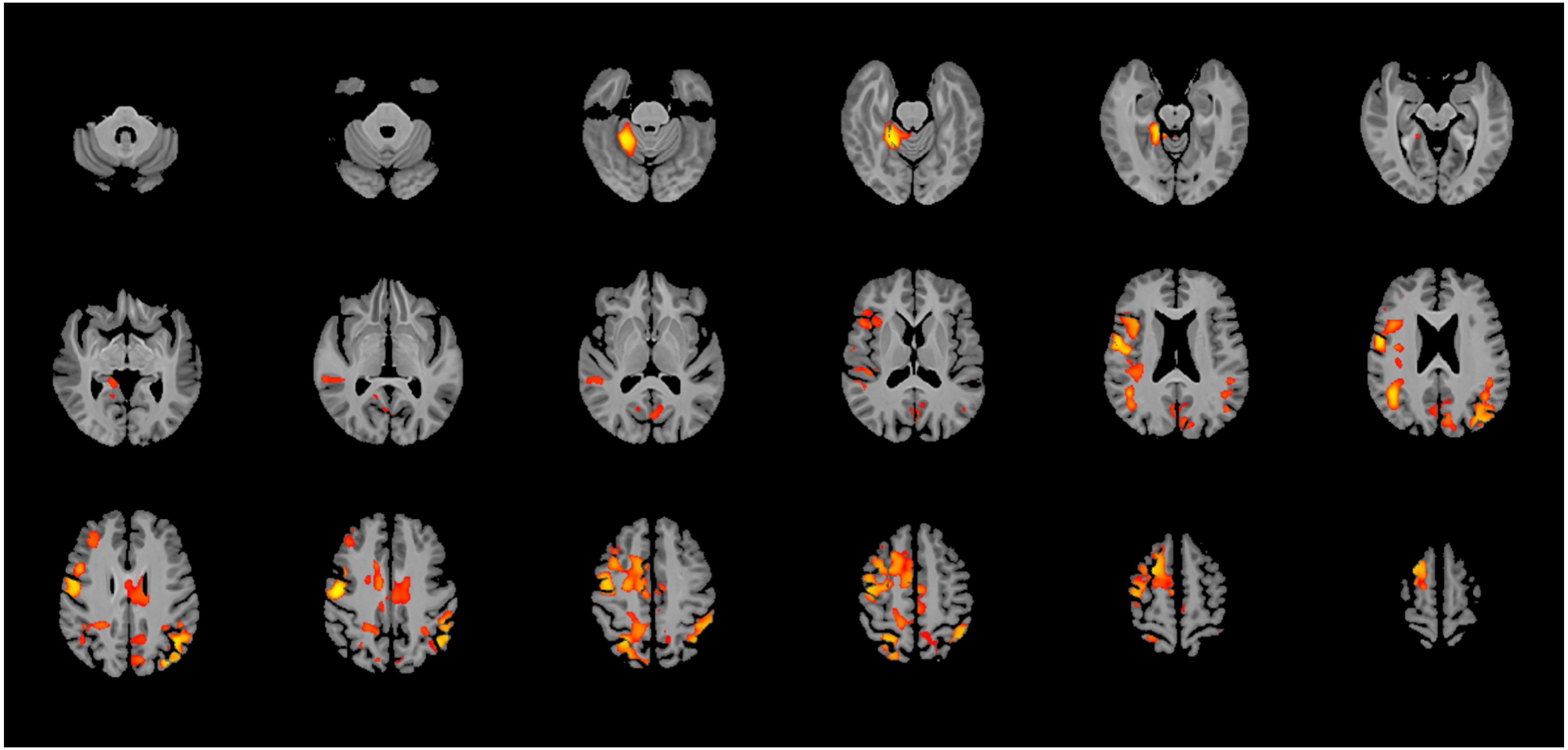

Supplementary Figure 7. Whole-brain QSM for disease status Clusters where absolute susceptibility is significantly higher in patients with active than stable disease are shown for FWE-corrected P values < 0.05. Clusters are overlaid onto the study-wise template. Axial slices are shown.

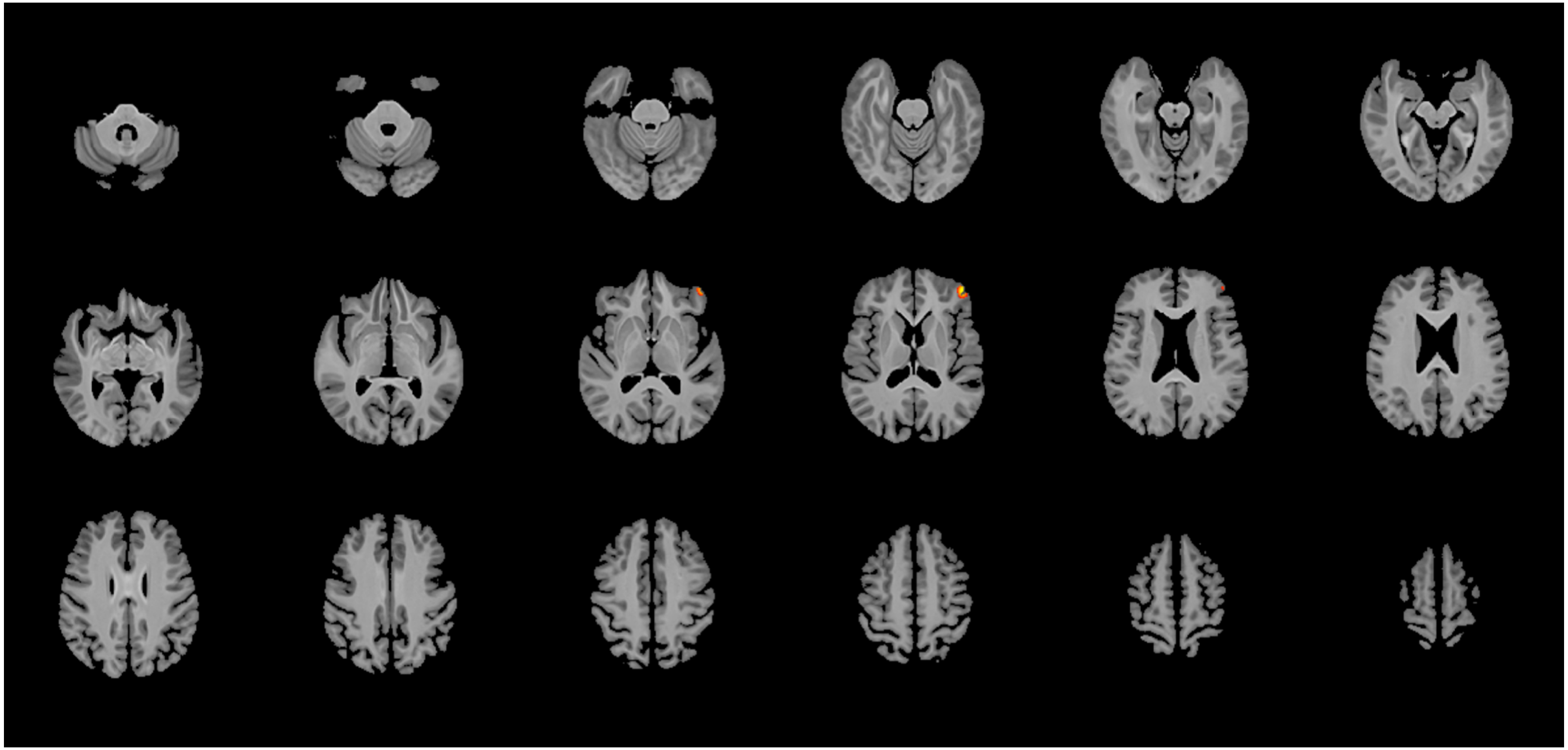

Supplement: awab274_Supplementary_Data [file awab274_supplementary_data.pdf]
